# Fraction n-Butanol of Radix Notoginseng Protects PC12 Cells from A*β*_25–35_-Induced Cytotoxicity and Alleviates Cognitive Deficits in SAMP8 Mice by Attenuating Oxidative Stress and A*β* Accumulation

**DOI:** 10.1155/2017/8469754

**Published:** 2017-10-03

**Authors:** Jin-Lan Huang, Ying-Qin Feng, Li-Ru Bai, Mei-Chun Qin, Zhe-Hao Xu, Zhao-Rong Liu, Wen-Bing Chen, Deng-Pan Wu, Zhen-Guo Zhong

**Affiliations:** ^1^Jiangsu Key Laboratory of New Drug Research and Clinical Pharmacy, Pharmacy School, Xuzhou Medical University, Xuzhou, Jiangsu 221004, China; ^2^Department of Pharmacology, Pharmacy School, Xuzhou Medical University, Xuzhou, Jiangsu 221004, China; ^3^Department of Pharmacy, Liuzhou Traditional Chinese Medical Hospital, Liuzhou, Guangxi 545001, China; ^4^Scientific Research Center of Traditional Chinese Medicine, Guangxi University of Chinese Medicine, Nanning, Guangxi 530200, China

## Abstract

Chinese medicine has been used for Alzheimer's disease (AD) treatment for thousands of years with more effective and fewer side effects. Therefore, developing effective potential candidates from Chinese medicine against AD would be considered as critical and efficient therapy for AD treatment. This study was designed to evaluate the neuronal protective effect of fraction n-butanol (NB) of Radix Notoginseng on A*β*_25–35_-induced PC12 cells, explore the effect of the tested fraction on spatial learning and memory, and characterize the impacts of fraction NB on antioxidant enzymes, A*β* production, and APP and BACE1 expressions. The results revealed that fraction NB could promote proliferation of PC12 cells and protect and rescue PC12 cells from A*β*_25–35_-induced cell death. Moreover, fraction NB could improve spatial learning and memory impairments of senescence-accelerated prone8 (SAMP8) mice and attenuate oxidative stress and reduce the production of A*β* by inhibiting the expressions of APP and BACE1 in the brains of SAMP8 mice. The result of single dose acute toxicity assay showed that fraction NB had a mild toxicity in vivo. The pronounced actions against AD and in vivo low toxicity of fraction NB suggest that fraction NB may be a useful alternative to the current AD treatment.

## 1. Introduction

Alzheimer's disease (AD), the most prevalent cause of dementia worldwide, is defined neuropathologically by the deposition of amyloid-*β* peptides (A*β*) into senile plaques in the extracellular space, neuronal cell death induced by oxidative stress, and intracellular formation of neurofibrillary tangles. The senile plaque is mainly composed of A*β* with length of 40 or 42 residues. A*β* is generated from *β*-amyloid precursor protein (APP) firstly cleaved by *β*-secretase (*β*-APP cleaving enzyme 1, BACE1) and then by *γ*-secretase. Abnormal accumulation of extracellular A*β* in amyloid plaques causes neuronal damage, impairment of cognitive function, and ultimately the development of dementia [1]. Furthermore, growing evidence suggests that oxidative stress plays important roles in A*β* deposition and cognitive dysfunction of AD. It has been established that A*β* induces oxidative stress and oxidative stress in turn enhances the A*β* production and aggregation, which promotes neuronal degeneration and death, resulting in cognitive function impairment [2]. Antioxidant treatments in the early stage of AD pathogenesis were able to alleviate the cognitive function impairment and reduce A*β* accumulation in AD [3]. Accordingly, identification of potential protective candidates to attenuate oxidative stress and eliminate excessive accumulation of A*β* and ultimately improve cognitive function impairment is considered as an important strategy for AD treatment.

Radix Notoginseng, a widely used traditional Chinese medicine known as Sanqi or Tianqi in China, is the root of* Panax notoginseng *(Burk.) F. H. Chen belonging to the genus* Panax*, family Araliaceae. Studies have reported that Radix Notoginseng has plenty of pharmacological properties including antioxidant, antiatherosclerotic, antitumour, and haemostatic activities [4]. Recent studies have demonstrated that fraction n-butanol (NB) of Radix Notoginseng has antiproliferative effects on human colorectal cancer cells [5], inhibitory effects on osteoclastogenesis in LPS-activated RAW264.7 cells [6], and anti-inflammatory effects on collagen-induced arthritic mice [7]. However, it is unclear whether fraction NB has antagonistic effect on AD.

We and others reported that fraction NB extracted from Radix Notoginseng contained a large proportion of ginsenoside Rb1, Rg1, and notoginsenoside R1 [5, 8]. It has been established that ginsenoside Rb1, Rg1, and notoginsenoside R1 have abilities to suppress oxidative stress and attenuate A*β*-induced neuronal damage [9–11]. Thus, it is reasonable to hypothesize that fraction NB might have an effect against AD.

Therefore, this study was designed to evaluate the neuronal protective effect of fraction NB extracted from Radix Notoginseng on A*β*_25–35_-induced PC12 cells. Moreover, 3-month-old senescence-accelerated prone8 (SAMP8) mice were intragastrically given fraction NB for 8 consecutive weeks to explore the effect of the tested fraction on spatial learning and memory and characterize the impacts of fraction NB on antioxidant enzymes, A*β* production, and APP and BACE1 expressions, thereby providing a new opportunity for research in regard to the pharmaceutical prevention and treatment of AD.

## 2. Material and Methods

### 2.1. Chemicals

Radix Notoginseng were collected in Yulin city, Guangxi Zhuang Autonomous Region, China, and verified by Professor Wei songji from Guangxi University of Chinese Medicine. The extract condition of fraction NB was performed according to our previous study [8]. Briefly, air-dried and powdered Radix Notoginseng roots (4.5 kg) were conducted with 95% and 60% ethanol (each 4000 mL) by percolating at room temperature for a week to give total ethanol extract (1400 g). The extract was dissolved in water and then extracted with petroleum ether (PE), acetic ether (AE), and then water-saturated n-butanol (NB) to obtain fractions PE (24.4 g), AE (213.0 g), and NB (600.0 g).

### 2.2. Reagents

Methyl thiazolyl tetrazolium (MTT) was obtained from Amresco (Ohio, USA). Huperzine A (Hup A) was from Zhejiang Zhenyuan Pharmaceutical Co., Ltd. (Zhejiang, China). The SOD and GSH-PX activity detection kits were purchased from Nanjing Jiancheng Bioengineering Institute (Nanjing, China). TIANScript RT Kit was from Tiangen Biotech Co. Ltd. (Beijing, China). A*β*_25–35_ was from Gibco (New York, USA). All other reagents were from Sigma-Aldrich unless stated otherwise.

### 2.3. Cell Culture

PC12 cell line purchased from Shanghai Biological Institute was suspended in RMPI-1640 (Gibco, USA) containing 5% fetal bovine serum (FBS) (Hyclone, USA), 10% horse serum (Gibco, USA), 100 *μ*g/mL streptomycin, and 100 U/mL penicillin in 5% CO_2_ at 37°C.

### 2.4. Animals, Drug Treatment, and Tissue Preparation

It has been established that increased oxidative stress and A*β* immunoreactive granular structures were found in SAMP8 mice as young as 2 months of age. Remarkable memory deficits, apparent reduction in the activity of antioxidant enzymes such as superoxide dismutase (SOD) and glutathione peroxidase (GSH-PX), and increased amyloid plaque deposition were found in 5-month-old SAMP8 mice [12]. Thus, sixty 3-month-old SAMP8 and twelve senescence-accelerated-resistant (SAMR1) (3 months old, control strain of SAMP8) pathogen- and virus-free mice were obtained from Tianjin University of Traditional Chinese Medicine (Tianjin, China). SAMP8 mice were randomly divided into five groups: model group, fraction NB high-dosage, fraction NB middle-dosage, and fraction NB low-dosage groups, and Hup A group. Since Hup A has been widely used for the treatment of AD owing to its roles in inhibiting acetylcholinesterase (AChE) activity, reducing A*β* accumulation, alleviating oxidative stress, and improving cognitive function [13, 14], thus Hup A was considered as a positive control in the study. SAMR1 mice were considered as the control group. The high-, middle-, and low-dosage groups were intragastrically given 300, 150, and 75 mg/kg of fraction NB, respectively, per day while Hup A group was treated with 0.3 mg/kg Hup A by gavage every day for 2 months. The middle dose of fraction NB was confirmed according to the dose of Radix Notoginseng used in clinics and the dose of Hup A was determined on the basis of our previous study [15]. The same volume of distilled water was given to the model and control groups. After examination of learning and memory, the mice were ethically sacrificed and the brains were excised for ELISA assay, activity detection, and real-time PCR assay. Animal care and experimental procedures were implemented according to the document “Guidance Suggestions for Caring for Laboratory Animals” produced by the Ministry of Science and Technology of China in 2006.

### 2.5. MTT Assay

PC12 cells were seeded into 96-well plates at a density of 10^4^ cells/mL for 24 h. For the detection of cell proliferation, cells were treated with fractions PE, AE, and NB at the concentrations of 10 *μ*g/mL, 20 *μ*g/mL, 40 *μ*g/mL, and 80 *μ*g/mL for 72 h, respectively. To evaluate the protective effect of fraction NB, the cells were treated with fraction NB at the concentrations of 10 *μ*g/mL, 20 *μ*g/mL, 40 *μ*g/mL, and 80 *μ*g/mL for 24 h before exposure to 10 *μ*M A*β*_25–35_, and the cells were then incubated with A*β*_25–35_ and different concentrations of fraction NB for another 72 h. For assessing the therapeutic effect of fraction NB, the cells were incubated with 10 *μ*M A*β*_25–35_ for 24 h. After the incubation, cells were exposed to fraction NB at the indicated concentrations for 72 h. After treatments, MTT assay was performed according to our previous study [16].

### 2.6. Single Dose Acute Toxicity

A single dose acute toxicity study was performed to assess the acute toxicity of fraction NB. The toxicity protocol was described previously [17, 18] and approved by the State Food and Drug Administration of China. Briefly, healthy Kunming-strain mice (*n* = 20, 18–22 g, male) from Guangxi University of Chinese Medicine were intragastrically given once fraction NB at 194.5 g/kg. Untreated mice were used as a negative control. A bolus dose of more than 194.5 g/kg of fraction NB was not attempted due to the limited solubility of fraction NB in the vehicle solution. After administration, mice overall health was monitored and body weight was detected on days 7 and 14. On day 14, fraction NB-treated mice and untreated mice were ethically sacrificed after anesthesia. The organs including liver, heart, kidney, and spleen were excised and weighted. The relative organ weight was calculated. Relative organ weight = organ weight (g)/body weight (g) × 100%. Moreover, the maximal tolerant dose, which is the dose of Radix Notoginseng confirmed by the maximal dosage of fraction NB, was calculated and the clinical daily dose of Radix Notoginseng was determined based on the dosage used in clinics. Since maximal tolerant multiple (MTM) is one of the parameters widely used to assess the acute toxicity of new drugs of traditional Chinese medicine [17–19], MTM was calculated to assess the toxicity of fraction NB according to the following formula [18, 19]:

(1) MTM=maximal  tolerant  dosegaverage  body  weight  of  mouse20 g÷clinical  daily  dose  of  Radix  Notoginsenggaverage  human  body  weight60 kg.

### 2.7. Morris Water Maze Test

The capacity for spatial learning and memory was carried out using Morris water maze (MWM) test as previously described [20]. The capacity of spatial learning was examined by place navigation test. The mice were subjected to four consecutive daily training trials. Before the trials, the mice were subjected to a water maze task in a swimming pool for 2 minutes in the absence of the platform to adapt to the environment. In each trial, the mice were randomly placed to the water at one of four start locations and trained to locate the hidden platform with a maximum trial time of 60 seconds and an interval of approximately 30 seconds. The mice were allowed to stay on the platform for 3 seconds before starting the next trial from another location. The platform was located in the same position for all trials. The time to reach the platform (escape latency) and the percentage of time in the target quadrant were recorded. On day 5, a spatial probe test was employed to detect the capacity of spatial memory. In the test, the mice were subjected to a water pool without the hidden platform and started at the start location in the quadrant opposite to the quadrant where the former platform was placed. The percentage of time spent in the former platform quadrant was recorded.

### 2.8. SOD and GSH-PX Activity Detection

SOD and GSH-PX activities were measured in accordance with the activity assay kit instructions of SOD and GSH-PX, respectively. Action buffers were mixed with the supernatants of tissue samples and incubated at 37°C. A visible spectrophotometer was performed to detect the optical densities of the mixtures at 550 nm and 405 nm for SOD and GSH-PX activities, respectively. Experimental data for the activities were expressed as the units per mg protein sample.

### 2.9. Enzyme-Linked Immunosorbent Assay (ELISA)

After the behavioral tests, the mice were sacrificed. The brains were homogenized (1 : 10 w/v) in cold normal saline and the supernatants were decanted for the measurement of total A*β* level using a commercially available high-sensitive ELISA kit (CUSABIO Biotech Co. Ltd., China) following manufacturer instructions. Each sample was measured in duplicate. Data are represented as nanogram (ng) per mL.

### 2.10. mRNA Extraction, Reverse Transcription, and Real-Time PCR

2 *μ*g total RNA, 4 *μ*L 5 × RT Buffer, 1 *μ*L RT Enzyme Mix, 1 *μ*L Primer Mix, and RNase-free water were contained in the reaction system of reverse transcription. Real-time PCR reaction was performed according to our previous study [15]. The primer sequences were as follows: BACE1 (307 bp), sense (5′-CTGTTATGGGAGCCGTCATC-3′), antisense (5′-GCAAAGTCATCGTGCTGGT-3′); APP (314 bp), sense (5′-GGAAGCAGCCAATGAGAGAC-3′), antisense (5′-AGAGCAGGGACAGAGACTGG-3′); ACTB (263 bp), sense (GAGACCTTCAACACCCCAGC), antisense (ATGTCACGCACGATTTCCC). The relative gene expression was calculated by 2^–ΔΔCp^ method, where ΔΔCp = ΔCp^treatment^ − ΔCp^control^ and ΔCp = Cp^target  gene^ − Cp^*A*^CT^*B*^gene^^ [15, 21].

### 2.11. Statistical Analysis

Experimental data in each group were presented as mean ± SD. Analysis of variance was performed with SPSS software for windows 13.0. *P* < 0.05 was considered statistically significant.

## 3. Results

### 3.1. Effects of Fractions Isolated from Radix Notoginseng on Proliferation in PC12 Cells

**Figure 1:**
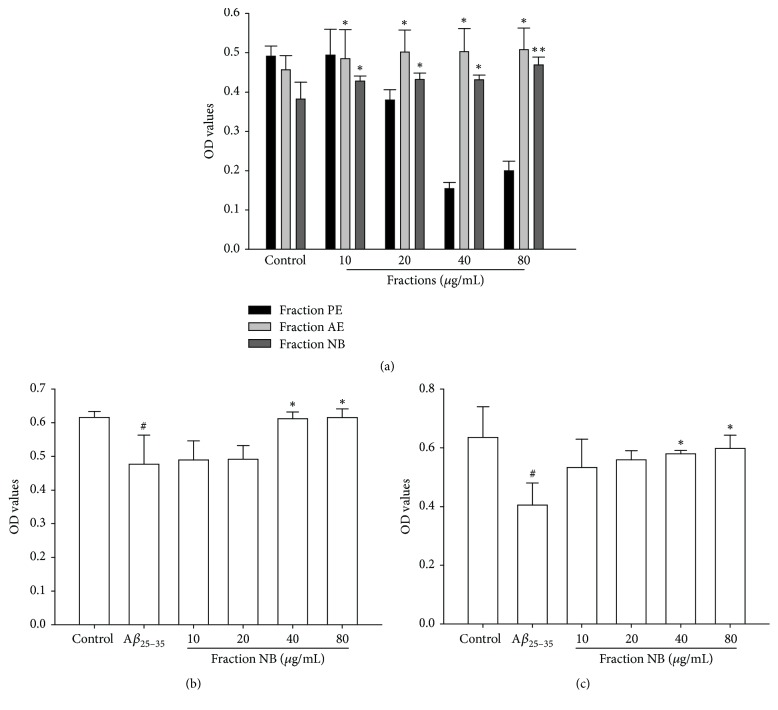
Effect of fraction NB on proliferation and A*β*_25–35_ induced cytotoxicity in PC12 cells. (a) Effects of the fractions isolated from Radix Notoginseng on proliferation of PC12 cells. (b) Cells were pretreated with fraction NB (10–80 *μ*g/mL) for 24 h before being incubated with 10 *μ*M A*β*_25–35_ and OD values were measured using the MTT assay. (c) Cells were with 10 *μ*M A*β*_25–35_ for 24 h and then posttreated with 10–80 *μ*g/mL fraction NB for 72 h and OD values were measured using the MTT assay. The data in different groups were expressed as the mean ± SD from 3 experiments. Statistical difference between groups was assessed by *t*-test using the SPSS 13.0 software. ^#^*P* < 0.05, versus the control group; ^*∗*^*P* < 0.05, ^*∗∗*^*P* < 0.01 versus A*β*_25–35_-treated group.

Firstly, for assessing the effects of the fractions isolated from Radix Notoginseng on cell proliferation, we treated PC12 cells with different concentrations of fraction PE, AE, or NB extracted from Radix Notoginseng for 72 h. MTT assay was performed to assess the effect of the fractions on proliferation of PC12 cells. As shown in Figure 1(a), the increased proliferative ability was observed in fractions AE- and NB-treated cells, while a sharp reduction in the proliferation was shown in fraction PE-treated cells. It should be noted that fraction NB-treated cells showed a higher proliferative ability than fraction AE-treated cells (Figure 1(a)), indicating that fraction NB may have potential to promote PC12 cells proliferation.

### 3.2. Effect of Fraction NB on A*β*_25–35_ Induced Cytotoxicity in PC12 Cells

Plenty of studies showed that A*β*-induced cytotoxicity in PC12 cells was a common and reliable cellular toxicity model for AD related studies in vitro [22]. To evaluate the protective effect of fraction NB, PC12 cells were pretreated with fraction NB for 24 h prior to A*β*_25–35_ incubation and then treated with the tested fraction for 72 h. Results of MTT assay showed that exposure of cells to 10 *μ*M A*β*_25–35_ for 24 h led to a significant decrease in cell viability (Figure 1(b)), while pretreatment with 40 *μ*g/mL and 80 *μ*g/mL fraction NB significantly suppressed A*β*_25–35_-induced cell death (Figure 1(b)). In order to clarify whether fraction NB could rescue the cells from A*β*_25–35_-induced cytotoxicity, PC12 cells were incubated with A*β*_25–35_ for 24 h and then posttreated with fraction NB at indicated concentrations for 72 h. The results indicated that fraction NB at 40 *μ*g/mL and 80 *μ*g/mL could remarkably attenuate A*β*_25–35_-induced cell death (Figure 1(c)). These results demonstrate that fraction NB holds the potential not only to protect but also to rescue PC12 cells from A*β*_25–35_-induced cell death.

### 3.3. Assessment of Acute Toxicity of Fraction NB Using Single Dose Acute Assay

**Figure 2:**
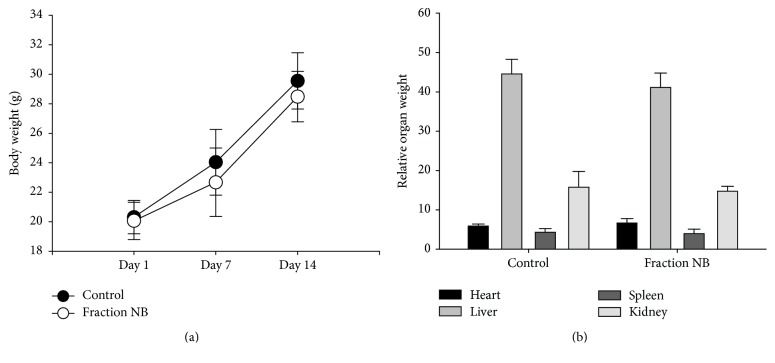
Assessment of acute toxicity of fraction NB using single dose acute assay. (a) Body weights of fraction NB-treated and control mice. Mice were given fraction NB at a single dose of 194.5 g/kg. Body weight of each mouse was recorded on days 1, 7, and 14 after treatment. (b) Relative organ weights of liver, heart, spleen, and kidney. On day 14 after treatment, mice were ethically sacrificed and heart, liver, spleen, and kidney were excised. Relative organ weights of liver, heart, spleen, and kidney were calculated. Data in each experiment represent mean ± SD from 20 independent samples. Statistically significant differences were calculated by one-way ANOVA using the SPSS 13.0 software.

Due to the limited solubility of fraction NB in the vehicle solution, a dose of 194.5 g/kg was considered as a maximal dose. In order to assess the acute toxicity of fraction NB at this dosage, mice were intragastrically given a bolus dose of 194.5 g/kg of fraction NB. As shown in Figure 2, the body weights of fraction NB-treated and control mice gained from 20.06 ± 1.26 g and 20.32 ± 1.13 g to 28.49 ± 1.71 g and 29.56 ± 1.90 g, respectively, and the body weight of fraction NB-treated group on days 1, 7 and 14 did not have significant difference compared to the control group. Moreover, the changes of relative organ weights of liver, heart, kidney, and spleen in fraction NB-treated group did not show any difference when compared with the control group (Figure 2). Additionally, the acute toxicity of fraction NB was also estimated using MTM parameter. MTM is the multiple of maximal tolerant dose of mice divided by clinical daily dose of humans as described in in Material and Methods. It is generally believed that the tested drug is considered safe if the maximal tolerant dose of mice is more than 100 times as high as the clinical daily dose of humans (MTM ≥ 100) [18, 19]. The result showed that the maximal tolerant dose of mice is 972 times as high as the clinical daily dose of humans (MTM = 972), suggesting a low toxicity of fraction NB. Together, the above results indicate that fraction NB has a mild toxicity in vivo.

### 3.4. Effect of Fraction NB on Learning and Memory Abilities of SAMP8 Mice in the Morris Water Maze

**Figure 3:**
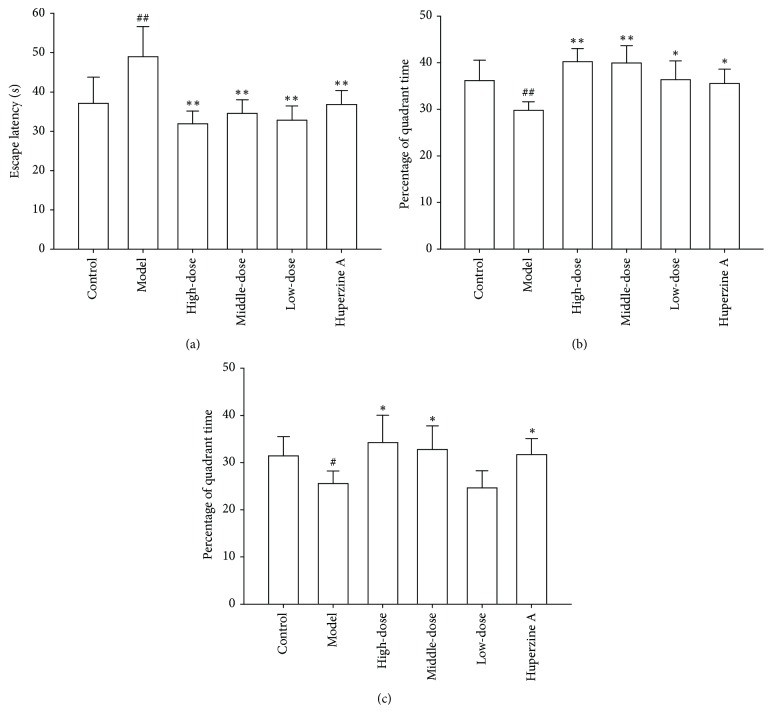
Effect of fraction NB on learning and memory abilities of SAMP8 in the MWM test. After treatment with different concentrations of fraction NB, the learning and memory abilities of SAMP8 mice were detected using MWM. ((a) and (b)) The average escape latency and percentage of time in the target quadrant of four-day training, respectively. (c) The percentage of time spent in the target quadrant of each mouse on day 5. Data in each experiment represent mean ± SD from 10–12 independent samples. Statistically significant differences were calculated by one-way ANOVA using the SPSS 13.0 software. ^#^*P* < 0.05, ^##^*P* < 0.01 versus the control group; ^*∗*^*P* < 0.05, ^*∗∗*^*P* < 0.01 versus the model group.

The cognitive function was evaluated using MWM test. In the place navigation test, the average escape latency and percentage of time in the target quadrant of four-day training were calculated. As shown in Figure 3, the mean escape latency of four days of learning in control group and fraction NB-treated groups was significantly shorter than that in the model group, while the percentages of quadrant time increased in the control group and fraction NB-treated groups compared to the model group. In the probe trial of MWM study, the percentage of time spent in the target quadrant of each mouse was recorded to evaluate the learning and consolidation of the location of target quadrant during the four days of training. As illustrated in Figure 3, the percentage of quadrant time in the model group significantly decreased when compared to the control group. Nevertheless, after SAMP8 mice were treated with different concentrations of fraction NB, the percentage of quadrant time markedly increased (Figure 3). These results suggest that fraction NB can improve spatial learning and memory impairment of SAMP8 mice.

### 3.5. Effect of Fraction NB on SOD and GSH-PX Activities in the Brains of SAMP8 Mice

**Figure 4:**
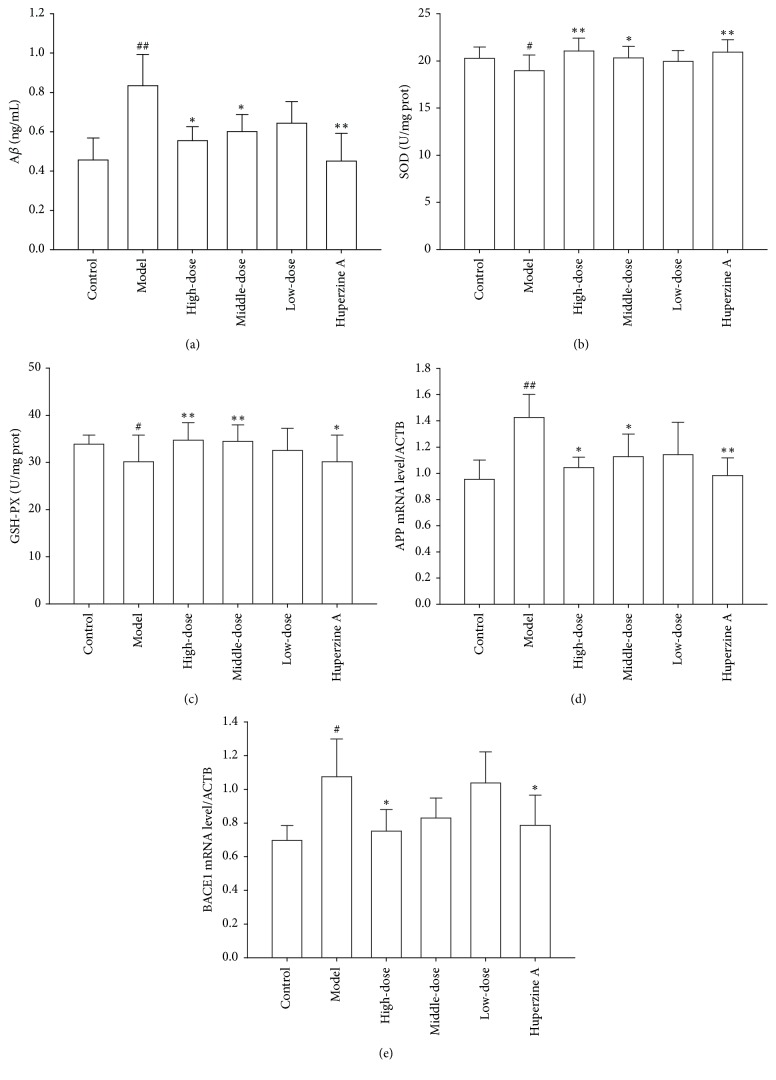
(a) Effect of fraction NB on A*β* accumulation in SAMP8 mice brains. After treatment with fraction NB, the level of A*β* in the brains of SAMP8 mice was detected using ELISA assay. ((b) and (c)) Effect of fraction NB on SOD and GSH-PX activities in the brains of SAMP8 mice. After treatment with fraction NB, the activities of SOD and GSH-PX were measured using activity detection kit. ((d) and (e)) Effect of fraction NB on APP and BACE1 mRNA expressions. Data in each experiment represent mean ± SD from 4-5 independent samples. Statistically significant differences were calculated by one-way ANOVA using the SPSS 13.0 software. ^#^*P* < 0.05, ^##^*P* < 0.01, versus the control group; ^*∗*^*P* < 0.05, ^*∗∗*^*P* < 0.01 versus the model group.

To investigate whether fraction NB could attenuate oxidative stress in the brains of SAMP8 mice, the activities of antioxidant enzymes such as SOD and GSH-PX were detected using activity detection kit. As illustrated in Figures 4(b) and 4(c), the activities of SOD and GSH-PX were remarkably lower in the model group than in the control group. After SAMP8 mice were treated with high- and middle-dosage fraction NB, the activities of SOD and GSH-PX were significantly increased. These results demonstrate that fraction NB has the ability to attenuate oxidative stress in the brains of SAMP8 mice.

### 3.6. Effect of Fraction NB on A*β* Accumulation in SAMP8 Mice Brains

To determine whether fraction NB had an effect on A*β* production, ELISA assay was used to measure the level of A*β* in the brains of SAMP8 mice after the mice were intragastrically administrated with fraction NB. As shown in Figure 4(a), the levels of A*β* in the control and fraction NB high- and middle-dosage groups were significantly lower than that in the model group, indicating that fraction NB suppresses the accumulation of A*β* in the brains of SAMP8 mice.

### 3.7. Effect of Fraction NB on APP and BACE1 mRNA Expressions

Since A*β* is generated from APP through cleavages by BACE1, a rate-limiting enzyme of A*β* production [23], we examined the effects of fraction NB on mRNA levels of APP and BACE1 in the brains of SAMP8 mice. As shown in Figures 4(d) and 4(e), the mRNA levels of APP and BACE1 in the control group were significantly lower than in the model group. After SAMP8 mice were treated with high- and middle-dosage fraction NB, APP mRNA expression was remarkably decreased. Meanwhile, high-dosage fraction NB markedly suppressed the BACE1 mRNA expression of SAMP8 mice. These results suggest that fraction NB treatment has the capability to inhibit APP and BACE1 mRNA expressions.

## 4. Discussion

Alzheimer's disease (AD), the most prevalent cause of dementia worldwide, affects millions of people every year. Unfortunately, up to now, there is no effective treatment for the disease. Available therapies only offer small symptomatic relief without preventing the progression of the disease and with considerable side effects. Chinese herbal medicine has been used for AD treatment for thousands of years with more effective and fewer side effects. Therefore, developing effective potential candidates from Chinese herbal medicine against AD would be considered as critical and efficient therapy for AD treatment. In the present study, we extracted fractions PE, AE, and NB from Radix Notoginseng and evaluated their effects on proliferation of PC12 cells using MTT assay. It was observed that fraction NB had a pronounced ability to promote proliferation of PC12 cells (Figure 1(a)). Moreover, the effects of fraction NB on A*β*_25–35_-induced cytotoxicity in PC12 cells were examined. Consistent with other reports, we observed that the viability of cells exposed to A*β*_25–35_ was markedly inhibited when compared to the controls (Figure 1(b)). Pretreatment or posttreatment with fraction NB significantly suppressed A*β*_25–35_-induced cytotoxicity (Figure 1(c)), indicating that fraction NB holds the potential not only to protect but also to rescue PC12 cells from A*β*_25–35_-induced cell death.

The acute toxicity of fraction NB was assessed using single dose acute toxicity assay approved by the State Food and Drug Administration of China. After mice were intragastrically given a single dose of 194.5 g/kg, a maximal dose due to the limited solubility, the body weights of fraction NB-treated and control mice gained from 20.06 ± 1.26 g and 20.32 ± 1.13 g to 28.49 ± 1.71 g and 29.56 ± 1.90 g, respectively, and the changes of body weight of fraction NB-treated group on days 1, 7, and 14 did not show significant difference compared to the control group (Figure 2). Meanwhile, the relative organ weights of liver, heart, kidney, and spleen in fraction NB-treated group showed no significant difference compared to the control group (Figure 2). In addition, since MTM ≥ 100 is adopted as the standard for the drug safety [18, 19], the result that the maximal tolerant dose of mice is 972 times as high as the clinical daily dose of humans (MTM = 972) indicates a low toxicity of fraction NB. Taken together, the above results suggest that fraction NB has a mild toxicity in vivo.

AD patients present progressive decline in cognitive function and personality impairment. In the early stage of AD, the patients manifest short-term memory loss, and in advanced stage, the patients manifest confusion, aggression, mood swings, long-term memory loss, and social isolation [24]. In order to assess the effect of fraction NB on spatial learning and memory function of SAMP8, all mice underwent MWM test after 8 weeks of drug administration. The results from the place navigation test revealed that the escape latency of fraction NB-treated mice was decreased, while percentages of time in the target quadrant in fraction NB-treated groups were remarkably increased when compared to the model group (Figure 3). The findings of the probe trial showed that treatment with fraction NB significantly increased the percentage of quadrant time (Figure 3). These results indicate that fraction NB possesses the ability to improve cognitive dysfunction in SAMP8 mice.

It has been established that oxidative stress plays important role in early AD stage [25]. Oxidative imbalance and resultant neuronal damage may promote neuronal degeneration and death, resulting in cognitive deficits [25]. The impaired antioxidant enzymes such as SOD and GSH-PX were found in postmortem brain tissue of AD patients [26]. In the study, the activities of SOD and GSH-PX in the brains of SAMP8 mice were examined, the results of which showed that a significant decrease in the activities of SOD and GSH-PX was found in the brains of SAMP8 mice when compared to the control SAMR1 mice (Figures 4(b) and 4(c)). These results are consistent with the trend of the data published in literatures [27]. Importantly, fraction NB treatment remarkably increased the activities of SOD and GSH-PX in the brains of SAMP8 mice, demonstrating an antioxidant capacity of fraction NB.

It is established that APP can be cleaved by *β*- and *γ*-secretases, leading to the generation of A*β*, and BACE1 is the *β*-secretase responsible for A*β* production [28]. Overproduction of A*β* in the brains of AD patients triggers the complicated pathological changes, ultimately leading to cognitive dysfunction in AD [29]. Our study demonstrates that fraction NB could significantly reduce the production of A*β* by inhibiting mRNA expressions of APP and BACE1 (Figures 4(a), 4(d), and 4(e)), indicating that inhibition of APP and BACE1 mRNA expressions may be one of the mechanisms of fraction NB precluding A*β* generation. However, whether fraction NB has effects on *γ*-secretase needs to be investigated in further study.

There are reports showing that oxidative stress contributes to A*β* generation either by downregulating the low-density lipoprotein receptor-related protein 1 (LRP-1) and upregulating the receptor for advanced glycation end products (RAGE) or by increasing the activity of *β*- and *γ*-secretases [30]. A*β* in return activate free radical generation by the nicotinamide adenine dinucleotide phosphate (NADPH) oxidase complex, leading to increased production of ROS [31]. Overproduction of ROS and abnormal A*β* deposition in the brains of AD patients ultimately cause neuronal apoptosis, giving rise to cognitive impairment [2]. Therefore, pharmacologic strategies designed to suppress both oxidative stress and A*β* generation may be more beneficial to the prevention and treatment of AD. The findings that fraction NB could attenuate oxidative stress and reduce A*β* deposition, thereby improving cognitive dysfunction of SAMP8 mice, demonstrated that fraction NB may be a promising candidate for AD treatment.

## 5. Conclusion

Our results reported here demonstrate that fraction NB has a prominent ability to promote proliferation and protect A*β*_25–35_-induced neurotoxicity in PC12 cells and alleviates cognitive impairment in SAMP8 mice through attenuating oxidative stress and A*β* accumulation by inhibiting APP and BACE1 expressions. It is noteworthy that high dose of fraction NB has shown almost equally positive outcomes as compared to Hup A, which may result from the similar mechanism of action of fraction NB to Hup A [13, 14, 32]. Thus, the pronounced actions of fraction NB against AD and its low toxicity in vivo suggest that fraction NB may be a useful alternative to AD treatment. It should be noted, however, that the treatment of fraction NB was initiated in 3-month-old SAMP8 mice prior to the onset of AD-like pathology and functional impairments. Consequently, whether fraction NB could relieve impairments after the onset of AD symptoms and whether fraction NB can delay the onset or has preventive effects on AD should be investigated in further study.
